# Contact Investigation in Households of Patients with Tuberculosis in Hanoi, Vietnam: A Prospective Cohort Study

**DOI:** 10.1371/journal.pone.0049880

**Published:** 2012-11-15

**Authors:** Gregory James Fox, Nguyen Viet Nhung, Dinh Ngoc Sy, Luu Thi Lien, Nguyen Kim Cuong, Warwick John Britton, Guy Barrington Marks

**Affiliations:** 1 Woolcock Institute of Medical Research, Sydney, New South Wales, Australia; 2 Centenary Institute of Cancer Medicine and Cell Biology, Sydney, New South Wales, Australia; 3 Central Clinical School, Sydney Medical School, University of Sydney, Sydney, New South Wales, Australia; 4 National Lung Hospital, Hanoi, Vietnam; 5 Hanoi Lung Hospital, Hanoi, Vietnam; 6 Department of Tuberculosis, Hanoi Medical University, Hanoi, Vietnam; McGill University, Canada

## Abstract

**Setting:**

Existing tuberculosis control strategies in Vietnam are based on symptomatic patients attending health services for investigation. This approach has not resulted in substantial reductions in the prevalence of tuberculosis disease, despite the National Tuberculosis Program achieving high treatment completion rates. Alternative approaches are being considered.

**Objective:**

To determine the feasibility and yield of contact investigation in households of patients with smear positive pulmonary tuberculosis among household members of tuberculosis patients in Hanoi, Vietnam.

**Methods:**

Household contacts of patients with smear positive pulmonary tuberculosis were recruited at four urban and rural District Tuberculosis Units in Hanoi. Clinical and radiological screening was conducted at baseline, six months and 12 months. Sputum microscopy and culture was performed in contacts suspected of having tuberculosis. MIRU-VNTR molecular testing was used to compare the strains of patients and their contacts with disease.

**Results:**

Among 545 household contacts of 212 patients, four were diagnosed with tuberculosis at baseline (prevalence 734 cases per 100,000 persons, 95% CI 17–1451) and one was diagnosed with tuberculosis during the subsequent 12 months after initial screening (incidence 180 cases per 100,000 person-years, 95% CI 44–131). Two of these cases were culture positive for *M. tuberculosis* and both had identical or near-identical MIRU-VNTR strain types.

**Conclusion:**

Household contacts of patients with potentially infectious forms of tuberculosis have a high prevalence of disease. Household contact investigation is feasible in Vietnam. Further research is required to investigate its effectiveness.

## Background

Over 5 million people living in Asia develop tuberculosis (TB) each year, comprising 59% of the total global disease burden. [1] TB incidence is falling at an estimated 1.1% per year in South East Asia and 2.6% per year in the Western Pacific. These regions are likely to achieve the United Nations Millennium Development Goal of halving TB prevalence by 2014. [2] National TB control programs in Asia predominantly follow World Health Organization (WHO) recommendations that emphasise passive case detection, relying upon symptomatic patients to present to health care facilities for diagnosis. [3] This recommendation is predicated on the view that passive case finding is less expensive and patients who self-present are more likely to complete treatment. However, the case detection rate in South East Asia is estimated to be only 61% of the prevalence rate, indicating that this approach has resulted in a substantial proportion of infectious cases remaining undiagnosed. [4].

Contact investigation for TB is a form of active case detection entailing the systematic evaluation of the contacts of known TB patients to identify active disease or latent TB infection. The primary goal of contact investigation is the early diagnosis and treatment of contacts with disease, both interrupting ongoing transmission and reducing morbidity and mortality in affected individuals. This strategy may be worthwhile in contacts of patients with TB because they are at higher risk of TB than members of the general population. [5], [6].

Contact investigation has been widely implemented in high-income countries for decades. [7], [8], [9], [10] Recently there has been a growing interest in contact investigation in resource-limited settings as national programs seek new methods for improving case detection. Household contacts are frequently selected for contact investigation because they are likely to have prolonged exposure and share environmental risk factors with the index case and, therefore, are at greatest risk of TB infection and disease.

Vietnam is a south east Asian nation with a persistently high prevalence of TB despite reported treatment completion rates of over 90% since 1998. [1] A survey of 11,624 people in the capital, Hanoi, in 2003–4 found that the prevalence of smear positive TB was 146 per 100,000 people. [11] In 2006–7, a survey of 94,179 people aged 15 years and above found that the national prevalence of smear positive disease was 196 per 100,000 people. [12] Only 22% of prevalent cases were currently receiving treatment. Taken together, these findings suggest that passive case-detection is unlikely to be sufficient to control TB in Vietnam.

The primary aim of this research was to assess the feasibility of an integrated contact investigation program within the Vietnamese TB control program. It also aimed to determine the yield of contact investigation among household contacts of patients with smear positive pulmonary TB over the year following exposure.

## Methods

### Study Setting

The study was set in District Tuberculosis Units (DTUs) in one rural and three urban districts in Hanoi. The capital, with a population of 6.5 million, comprises 10 inner districts, 18 rural districts and one town. DTUs are the primary site for coordinating diagnosis and treatment of TB in each district. However, a significant proportion of cases are diagnosed at two lung disease hospitals and referred to DTUs for treatment. Treatment is provided free of charge by the National Tuberculosis Program (NTP) and is directly observed by a nominated family member with the support of commune health care workers.

### Study Participants

Household contacts of patients with smear positive pulmonary TB (‘index patients’) were enrolled between September 2009 and January 2011. Index patients were defined as the first person in the household to be diagnosed with TB. Eligible index cases were aged 16 years or over, had pulmonary TB with at least one sputum sample positive for acid fast bacilli (AFB) on direct smear microscopy [13] and had at least one other person living in the same household. Index patients were asked to submit two spot sputum samples for repeat direct smear and solid culture at the time of enrolment, and blood was collected for HIV testing using commercial kits. Household contacts of any age were eligible for inclusion if they lived in the same dwelling as the index patient during the two months prior to their diagnosis.

### Description of Intervention

At the time of enrolment, patients were asked to name all eligible household contacts. District health care workers explained the rationale for contact investigation and delivered health promotional material for patients to take home. Index patients were then encouraged to bring their household members to the clinics as soon as possible after their initial diagnosis.

Patients and contacts were given a small subsidy for expenses associated with attending the clinic. After initial screening, household contacts were given appointments to return to the clinic for repeat screening after 6, 12 and 24 months. Contacts were advised to re-present immediately if they developed symptoms of TB (that is, cough, haemoptysis or weight loss).

At each visit, contacts were interviewed about their current symptoms and risk factors for TB and were examined for signs of extra-pulmonary disease. A plain chest radiograph was performed at each visit unless the contact was pregnant. All chest radiographs were read twice, once by District staff and once by a radiologist at the metropolitan TB hospital.

Contacts received a telephone reminder to attend their scheduled follow-up appointments. Health care staff also used these telephone calls to educate participants about the benefits of contact investigation.

At each visit, contacts were classified as ‘suspects’ if they reported having cough or sputum for two weeks or more, haemoptysis within the last month or if staff identified any abnormalities on the chest radiograph. Suspects were asked to submit three sputum samples, including an early morning sample. Sputum samples were transported by motorbike to the central hospital for microbiologic testing. Investigations for extra-pulmonary TB were performed according to standard clinical practice.

Household contacts who were diagnosed with TB were treated at the DTU using a standard eight-month drug regimen. Contacts treated for TB also underwent HIV testing using commercial rapid diagnostic kits.

### Definitions of Study Outcomes

Smear positive pulmonary TB was defined by the presence of at least one positive smear in combination with an abnormal chest radiograph, or one positive smear plus a positive culture. Smear negative TB was diagnosed if contacts had radiographic changes consistent with TB, no response to broad-spectrum antibiotics and a response to anti-tuberculous drug treatment. Extra-pulmonary disease was defined based upon clinical assessment, investigation and the decision to commence treatment for TB. Treatment outcomes were assessed after one year.

### Bacteriological Testing

Sputum samples were examined using Ziehl-Neelsen smear microscopy and solid culture on Lowenstein-Jensen medium. Positive cultures were stored in a −20°C freezer for subsequent genotyping. Paired isolates from index patients and their contacts were subjected to 24-loci mycobacterial interspersed repetitive-unit variable-number tandem repeat (MIRU-VNTR) testing according to standard methods. [14] Where available, two culture isolates were tested for each contact and patient. Identical or closely related MIRU-VNTR patterns were considered suggestive of transmission between the index case and the contact whereas distinct patterns indicated that the contact was not infected by the putative index case.

### General Population Estimates of Disease Prevalence

The prevalence of TB in the general population was estimated by screening 3,556 randomly selected subjects from three inner city districts, nested within a recent national prevalence survey that has been previously reported. [12] This population served as a control group for household contacts in the present study.

### Statistical Methods and Ethical Approval

Statistical analyses were conducted using SAS (version 9.2; Cary Corp., NC). We described the clinical characteristics of index patients and contacts using simple frequencies and medians with interquartile ranges. The primary outcomes were the prevalence and incidence rate of disease. Odds ratios comparing disease in the study population to that of the general population were calculated and tested using Fisher’s exact test.

The study was approved by the Human Research Ethics Committee at the University of Sydney, and the Institutional Review Boards of the Vietnam Ministry of Health and the National Lung Hospital, Vietnam. Written informed consent was obtained from all participants, with parents providing written consent for child contacts less than 16 years of age, in accordance with ethics committee approval.

## Results

### Index Patients

**Figure 1:**
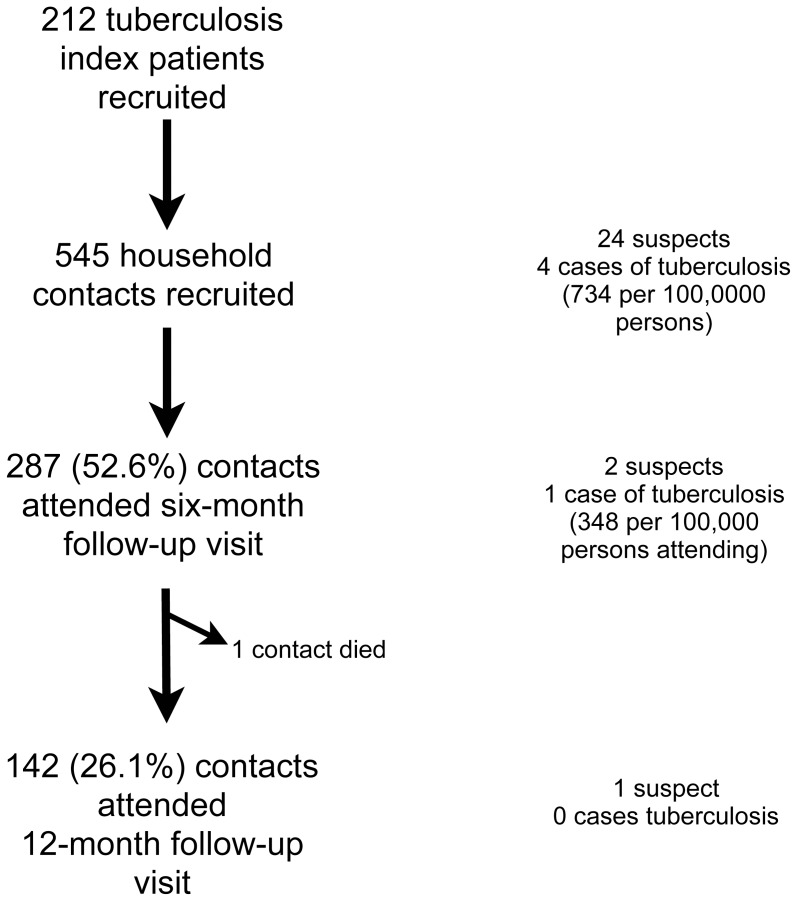
Summary of study outcomes.

Among 394 eligible patients who attended the four participating DTUs during the study period, 212 (54%) index patients were recruited, as shown in Figure 1. Index patients included 170 (80.2%) males and their mean age was 46.8 years (Table 1). There were 174 (91.1%) patients with cough of at least two weeks and 144 (77.0%) with sputum production for at least two weeks (Table 2). Twelve index patients (6.1%) tested positive for HIV.

**Table 1:** Summary of participant characteristics.

	Index patients, n (%)	Household contacts, n (%)
**Number**	212	545
**Male gender**	170 (80.2%)	220 (40.4%)
**Age, mean (yr)**	46.8 [31.8–57.1]	36.3 [34.7–37.9]
*** <5 years***	–	32 (5.9%)
*** 5–15 years***	–	60 (11.0%)
*** 16–24 years***	13 (6.1)	77 (14.1%)
*** 25–34 years***	50 (23.6)	93 (17.1%)
*** 35–44 years***	35 (16.5)	84 (15.4%)
*** 45–54 years***	42 (19.8)	91 (16.7%)
*** 55–64 years***	30 (14.2)	67 (12.3%)
*** > = 65 years***	31 (14.6)	29 (5.3%)
**Relationship to index patient**		
*** Spouse***	–	137 (25.2%)
*** Sibling***	–	47 (8.6%)
*** Child***	–	245 (45.0%)
*** Parent***	–	81 (14.9%)
*** Other***	–	34 (6.3%)

**Table 2:** Summary of the clinical characteristics of participants.

	Index patients, n (%)	Household contacts, n (%)
History of prior tuberculosis	20 (9.4%)	10 (2.0%)
Cough at least 2 weeks	174 (91.1%)	12 (2.2%)
Sputum at least 2 weeks	144 (77.0%)	9 (1.7%)
Any haemoptysis	34 (16.8%)	2 (0.37%)
Fever	132 (65.4%)	18 (3.4%)
HIV prevalence	12 (6.1%)	–

### Household Contacts

We recruited 545 out of the 657 (82.9%) household contacts identified by patients. Households had a mean of 2.6 contacts per household. Among contacts, 220 (40.4%) were male and their mean age was 36.3 years with 16.9% under the age of 16 years. Contacts reported spending an average of 10.8 hours per day (95% CI 10.2–11.4 hours/day) with the index patient during their infectious period. 65 (14.2%) contacts were current smokers, and on examination a BCG scar was identified in 120 (31.3%) contacts.

### Outcomes of Investigation at Baseline

At baseline, 24 subjects met the criteria for TB suspect, including 22 (92%) who had an abnormal chest X-ray, 16 (2.9%) who reported cough for two weeks or more, seven (1.3%) who had sputum for two weeks or more and one (0.2%) who had haemoptysis in the past month. Of these suspects, 13 were able to produce sputum for testing.

Four contacts were diagnosed with TB at the baseline assessment. Two of these had smear-positive pulmonary disease and two had extra-pulmonary disease. The prevalence of all forms of TB in contacts at baseline screening was 734 per 100,000 persons (95% CI 17–1451) and the prevalence of smear positive disease was 367 per 100,000 persons (95% CI 44–1319). Therefore, 136 people (95% CI 68–5,882) would need to be screened to identify one case. Table 3 presents the clinical characteristics of the contacts who were diagnosed with TB. The contact with spinal TB was diagnosed on the basis of clinical history, radiological findings and a response to TB therapy.

**Table 3:** Summary of contacts diagnosed with tuberculosis.

Gender	Age (y)	Time of diagnosis	Duration cough (weeks)	Duration sputum (weeks)	Haemoptysis	Xray findings	Sputum smear	Sputum culture	HIV result
Male	49	Baseline	0	0	No	Spinal TB	N/A	N/A	Negative
Male	34	Baseline	4	4	Yes	Typical for PTB	Positive	N/A*	Negative
Female	32	Baseline	4	1	No	Typical PTB with pleural TB	Negative	Negative	Negative
Male	43	Baseline	4	4	No	Typical for PTB	Positive	Positive	Negative
Female	15	6 months	2	2	No	Typical for PTB	Positive	Positive	Negative

### Outcomes of Repeat Screening and Follow-up

Among the 287 contacts attending the 6-month follow-up (44.0% of the initial cohort of contacts who had not been diagnosed with TB at the baseline assessment), one case of active TB disease was identified (180 per 100,000 persons in the initial cohort, or 348 per 100,000 persons attending the follow-up visit). One contact had died of a cause unrelated to TB before the 6-month visit. Among 142 (26% of initial cohort) who attended the 12-month follow-up visit, no new cases were identified. Four of the five contacts diagnosed with TB completed eight months of treatment. One contact defaulted from treatment after three months of family observed therapy, and then re-presented with relapsed disease nine months later. This individual then completed a course of treatment for TB.

### Estimate of TB Prevalence in the General Population

Among the 3,556 individuals screened in three urban districts in Hanoi during the NPS in 2006–7, the weighted prevalence of smear positive TB was 169 per 100,000 (95% CI 38–283). The difference in prevalence between the general population and the study population did not reach statistical significance.

### Outcomes of Culture and Molecular Typing

At least one sputum culture was positive for *Mycobacterium tuberculosis* in 135 (78.9%) of index patients for whom sputum culture results were available. Among contacts with TB, sputum culture was positive for *Mycobacterium tuberculosis* in two of three patients with positive sputum smears. The MIRU-VNTR patterns of isolates from one index patient-contact pair were identical. Isolates from the second index patient-contact pair were closely related but not identical, with a loss of three repeats in the isolate of the contact compared to that of the index patient. Both strains were of the Beijing lineage.

## Discussion

Increasing the detection of cases of TB is a high priority for Vietnam. The most efficient strategies are those implemented in high-risk populations that can be readily tested and treated. This district clinic-based contact investigation study showed the feasibility of contact investigation within urban and rural clinics of the government TB control program. It found a substantial prevalence of previously undiagnosed TB among household contacts. Our findings suggest that household contacts are a high-yield population for efficient screening to enhance TB case detection.

A key feature of this intervention was its integration within the national TB control program. Our strategy was implemented by existing district clinic staff, working within a system that delivers the majority of TB care throughout Vietnam. If a contact investigation program is to be sustainable on a large scale, it is essential that it be closely integrated with the existing TB control program. This integrated strategy, based on strong national and local leadership, is highly relevant to tuberculosis control programs in similar high-prevalence settings.

Another important aspect of our study was engagement of index patients to recruit members of their own household. This approach had several practical advantages. First, the approach draws upon the existing relationship that patients have with health care staff to increase the likelihood of cooperation from other family members. Second, our patient-directed approach ensures that patient consent is a prerequisite for family involvement, which addresses some of the ethical concerns surrounding patient confidentiality that arise in TB management. [15].

The prevalence of disease among contacts in our study of 0.73% is similar to that of many published studies,[16]–[23] although the point estimate was below the mean prevalence of 3.1% (95% CI 2.1%–4.5%, I^2^ 98.8%) reported in a recent meta-analysis of 71 studies from low-middle income countries [6]. That review found considerable variability between settings in the prevalence of TB disease in household contacts.

The use of chest X-ray [24] with double-reading of films will have enhanced the detection of TB suspects in the present study. On the other hand, the finding that only 54% of suspects were able to produce sputum may indicate that we have underestimated the prevalence of confirmed pulmonary TB disease. However, the majority of suspects who could not produce a sputum specimen were asymptomatic, suggesting that the observed radiological abnormalities may have been due to past, healed TB or non-tuberculous lung disease. In future case-finding programs, additional methods such as sputum induction should be considered as an adjunct to spontaneous sputum production in individuals who cannot produce sputum. [25], [26] This is likely to improve the yield of sputum samples, and improve the sensitivity of the screening approach, particularly in early disease.

It is also possible that we underestimated the number of incident cases owing to relatively low attendance at 6 and 12-month follow-up visits. We attempted to minimise default from follow-up by educating contacts about the key symptoms of disease at the initial visit, contacting them by telephone in advance of their 6 and 12 months appointments.

The incidence of TB remains high for several years after infection [27]. Hence, periodic screening during two years after exposure may be considered as a part of a contact investigation program and this approach is routine in some high-income countries [7]. In our study, we identified one new case at the six-month follow-up assessment. This is consistent with a recent meta-analysis that showed the incidence of TB among contacts was 1.5% (95% CI 0.9–2.4%, I^2^ 96.3%) during the first year after exposure. [12].

Effective screening requires high rates of attendance in contacts. In our study we improved compliance by drawing upon the therapeutic relationship between the patient and health staff during the eight months of treatment. Staff incorporated contact visits into the routine visits of patients, which is a relatively effective and low-cost strategy, in combination with delivering education about the early symptoms of TB to contacts.

In addition to issues of feasibility, the effectiveness of contact investigation compared to usual passive case-finding is an important issue for policy-makers. [28] Contact investigation aims to enable earlier identification of infectious patients, decreasing the period of infectiousness, thereby reducing ongoing transmission. As none of the contacts with TB in our study were known to have the disease at the time of diagnosis, it is likely that the intervention resulted in earlier diagnosis of the disease. However, we cannot predict what proportion would have eventually been diagnosed by ‘passive case finding’. In order to adequately determine effectiveness compared to usual care, it will be necessary to conduct a randomized controlled trial with matched control subjects that are not screened.

In order for active case-finding to reduce TB transmission, contacts not only need to be diagnosed, but also to complete treatment. [29] All contacts diagnosed with the disease commenced a standard 8-month course of TB treatment, and all eventually completed treatment. However, one individual initially defaulted from treatment, and subsequently recommenced a second course of therapy after a six month delay. These outcomes are similar to the 91% completion rate achieved in Vietnam among patients diagnosed by passive case-finding. [1].

Molecular testing of paired *M.tb* isolates in one index patient-contact pair in our study was consistent with direct transmission, based upon identical MIRU-VNTR patterns. In the second patient-contact pair, the isolate of the contact had three less bands in two loci compared to that of the index patient. While the genotype of *M.tb* in a population can lose or add repeats over time, [30] in a population with considerable strain heterogeneity, [31], [32] the loss of repeats at two separate loci probably indicates this is an independent strain which was acquired from another unrecognised patient. Other molecular epidemiology studies in low and high-prevalence settings have demonstrated that known index patients are not necessarily the source of infection in contacts. [33], [34], [35] A study from a low-prevalence setting found that 70% (95% CI 56–82%) of isolates from index patient-contact pairs shared identical strains. [34] In low prevalence settings, small differences in molecular typing can be useful marker of the presence of additional unknown source cases. [36] However, in high-prevalence settings, such as Vietnam, the risk of an individual being infected by an unrecognised patient is much greater and reactivation of longstanding infection is also more likely. Hence, while household exposure contributes to the risk of TB, it may not be the only source of infection. [37], [38] Nonetheless, household contacts should be considered a suitable target population for screening, because they often share other risk factors in addition to exposure to the known index patient.

There are a number of limitations in interpreting this study. The small sample size of this pilot study means that confidence intervals for outcomes are wide, precluding a conclusion about the difference among contacts and the general population. We also noted a substantial drop-off in attendance for six and twelve month follow-up visits. This varied considerably between Districts, with the lowest rates occurring in a district that lacked radiology facilities, and required contacts to travel more than three kilometres for an X-ray. While follow-up rates may have been higher if our study staff conducted routine home visits to promote screening, this approach would add considerable cost to the existing program and contacts would still need to attend a health care facility to have a chest X-ray. Finally, although child contacts were enrolled in the study, screening identified no suspects under the age of 15 years. This may reflect the actual prevalence, however given that the diagnosis of childhood TB is often challenging our contact investigation may have missed some cases, particularly of extra-pulmonary disease. Other strategies in addition to chest X-ray may be required in order to identify more effective strategies of identifying early disease children. Contact investigation programs can also provide a suitable framework for concurrently implementing isoniazid prophylactic therapy, for which there is evidence of both efficacy and cost-effectiveness. [39], [40], [41].

The role of contact investigation as a strategy for TB control in resource limited settings remains a subject of debate. [42], [43] Until recently WHO recommendations have emphasised the role of passive, rather than active, case-finding in TB control programs [44]. Recommendations for active case finding have been limited to contacts who are children and contacts of people living with HIV.[45]–[49] However, there is a growing interest in strategies that enhance case-finding for TB, reflected in new WHO guidelines for TB contact investigation in low and middle-income countries. [50] A recent survey identified 65 countries with a national TB contact investigation policy, although it did not evaluate how widely these policies were implemented. [51].

National programs should consider introducing active case finding if they have a program that is able to deliver therapy reliably, has high treatment completion rates and available resources to invest in enhanced case detection. Therefore, analysis of cost-effectiveness is essential to justify adding or diverting resources for widespread contact investigation. Further research, involving a comparable control population, should be performed before committing substantial resources to contact investigation. Furthermore, it is important to recognise that contact investigation will still only identify a minority of undiagnosed cases in the whole population, and should be considered in combination with other active case-finding strategies. This study has demonstrated the importance of pilot testing of contact investigation, to learn lessons about contact investigation in the local context in advance of its widespread implementation.

### Conclusion

TB remains a major public health challenge for Vietnam, despite a decade of high treatment completion rates. Enhancing case detection is now a key priority for the NTP, in order to reduce ongoing disease transmission in the community. This study shows that household contact investigation is a feasible strategy to achieve this goal. Further studies are required to assess its effectiveness and cost-effectiveness, particularly in resource limited settings.
